# SGK1 in Cancer: Biomarker and Drug Target

**DOI:** 10.3390/cancers14102385

**Published:** 2022-05-12

**Authors:** Jonas Cicenas, Edita Meskinyte-Kausiliene, Vigilijus Jukna, Arnas Rimkus, Jokubas Simkus, Diana Soderholm

**Affiliations:** 1 Proteomics Centre, Institute of Biochemistry, Vilnius University, Sauletekio al. 7, LT-10257 Vilnius, Lithuania; 2 MAP Kinase Resource, Bioinformatics, Melchiorstrasse 9, CH-3027 Bern, Switzerland; rimkus.arnas@gmail.com (A.R.); jo.simkus@gmail.com (J.S.); 3 Center of Animal Husbandry Selections, Breeding Values and Dissemination, Agriculture Academy, Vytautas Magnus University, Studentų g. 11, LT-53361 Akademija, Lithuania; edita.meskinyte-kausiliene@vdu.lt (E.M.-K.); vigilijus.jukna@asu.lt (V.J.); 4 Walker Art Center, 752 Vineland PI, Mineapolis, MN 55403, USA; d.soderholmphoto@gmail.com

**Keywords:** SGK1, SGK1 inhibitors, cancer, phosphorylation, biomarkers, kinases

## Abstract

Serum- and glucocorticoid-regulated kinases (SGKs) are members of the AGC family of serine/threonine kinases, consisting of three isoforms: SGK1, SGK2, and SGK3. SGK1 was initially cloned as a gene transcriptionally stimulated by serum and glucocorticoids in rat mammary tumor cells. It is upregulated in some cancers and downregulated in others. SGK1 increases tumor cell survival, adhesiveness, invasiveness, motility, and epithelial to mesenchymal transition. It stimulates tumor growth by mechanisms such as activation of K^+^ channels and Ca^2+^ channels, Na^+^/H^+^ exchanger, amino acid and glucose transporters, downregulation of Foxo3a and p53, and upregulation of β-catenin and NFκB. This chapter focuses on major aspects of SGK1 involvement in cancer, its use as biomarker as well as potential therapeutic target.

## 1. Introduction

**Figure 1:**
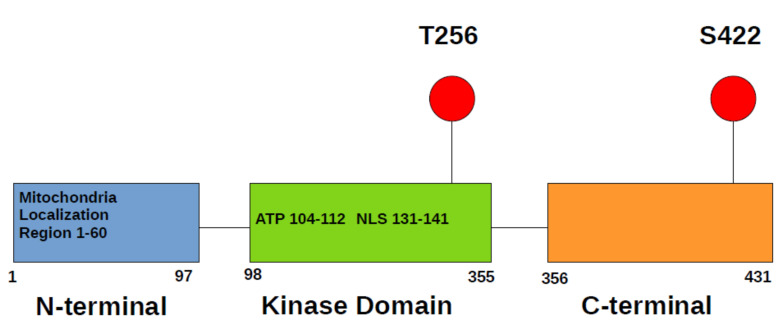
SGK1 domain structure. There is region necessary for mitochondrial localization in N-terminus and nuclear localization signal (NLS) in kinase domain. Kinase domain also contains ATP binding pocket. Most important activating phosphorylation sites are located in kinase domain and C-terminus.

The serum- and glucocorticoid-inducible kinase-1 (SGK1) was first discovered in rat tumor cells as a gene, transcriptionally regulated by serum and glucocorticoids [1] and, later, in human as gene upregulated by cell shrinkage [2]. The human SGK1gene is localized in chromosome 6q23. SGK1 is a member of AGC kinase family, which also includes kinases such as PKA, PKC, AKT, RSK, S6K, PDK1. As most AGC kinases, SGK1 consists of three domains: an N-terminal variable region, a catalytic domain, and the C-terminal tail (Figure 1). SGK1, SGK2, and SGK3 are closely related to AKT kinases; however, they lack lipid-binding PH domain.

**Figure 2:**
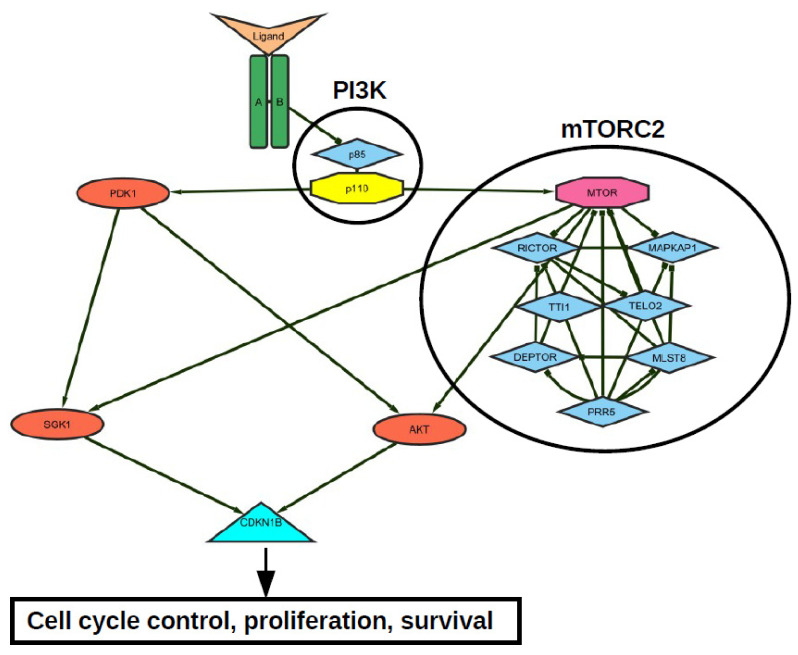
Representative receptor tyrosine kinase—mTORC2—PI3K—AKT—SGK1 signal network. Both AKT- and SGK-mediated CDKN1B (p27) T157 phosphorylation opposes p27-mediated G1 arrest and thus increases proliferation, survival and tumor growth.

Signaling proteins, which are involved in transcriptional SGK1 regulation, include p38 kinases, PKCs, RAF kinases, ERK1/2, ERK5, and PI3K. Molecules, such as cAMP, Ca^2+^, ROS, and nitric oxide are also involved [3,4]. Activation of SGK1 is triggered by signaling induced after cell stimulation with growth factors such as insulin, IGF1, or HGF. SGK1 function is strongly dependent on phosphorylation by mTOR. Through the mTOR-dependent hydrophobic motif phosphorylation on S422 [5], the kinase acquires an open conformation for phosphorylation and full activation by PDK1 which phosphorylates SGK1 at T256 [5]. PDK1-dependent activation of SGK1 is stimulated after the activation of PI3K by receptors for above mentioned growth factors (Figure 2).

NDRG1 and NDRG2 are known specific SGK1 targets, and there are other targets shared by the other SGK isoforms, AKTs and/or other kinases, including CARHSP1, CREB, ELK1, EnaC-alpha, ERK2, FBXW7, Fe65, FOXO3A, GDI1, GLUT4, Huntingtin, IkB-alpha, IKKB, iNOS, MEKK3, MKK4, Mnk1, NEDD4L, NHE3, nicastrin, p27Kip1, PIKFYVE, RICTOR, ROMK, SHANK2, SLC1A6, SRF, Tau, TRPV4, WNK1, and WNK4 [6]. Therefore, besides being involved in several downstream signaling events, as well as transcription regulation, SGK1 also upregulates a number of ion channels [7], stimulates a large amount of Na^+^, K^+^, 2Cl^−^ carriers [3,8], as well as some cytoskeletal proteins. The SGK1-dependent post-translational regulation of p53 demonstrated that SGK1 is able to regulate MDM2 [9,10]. Another target that is constantly emerging in the sgk1-mediated signaling is RANBP1. SGK1 regulates resistance to several chemotherapeutic drugs as well as cellular epigenetic rearrangement through the control of nuclear transport through RANBP1 [11,12]. Biological functions regulated by SGK1 include cell migration [13], cell survival [14], cell proliferation [14], cell volume regulation [3], degranulation [15], gastric acid secretion [16], glucose metabolism [3], hormone release [17], intestinal transport [17], muscle mass homeostasis [18], cytoskeleton arrangement [15], cellular K^+^ uptake [3,8], renal tubular K+ transport [19], renal tubular Na+ transport [20], inhibition of autophagy [5,21], and T-cell development [22].

## 2. SGK1 as Biomarker

**Figure 3:**
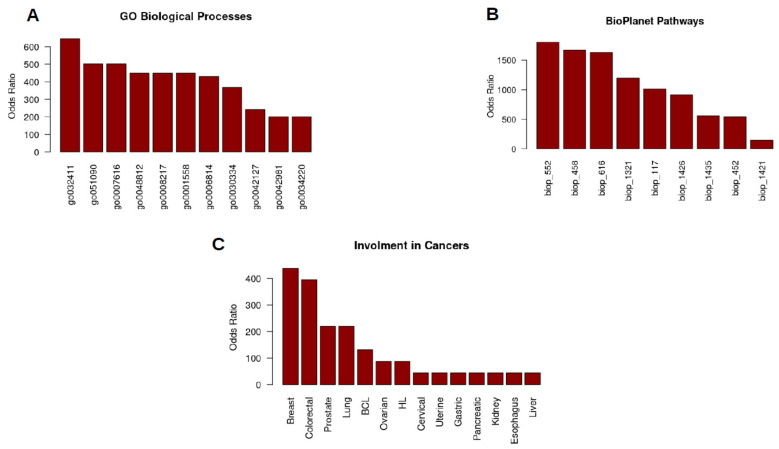
Involvement of SGK1 in major processes and cancers. (**A**) Most significant gene ontology biological processes in which SGK1 is involved. Positive regulation of transporter activity GO:0032411, regulation of DNA-binding transcription factor activity GO:0051090, long-term memory GO:0007616, neuron projection morphogenesis GO:0048812, regulation of blood pressure GO:0008217, regulation of cell growth GO:0001558, sodium ion transport GO:0006814, regulation of cell migration GO:0030334′ regulation of cell population proliferation GO:0042127, regulation of apoptotic process GO:0042981, ion transmembrane transport GO:0034220. (**B**) Involvement of SGK1 in most significant signaling pathways, calculated using BioPlanet data. FSH signaling pathway bioplanet_552, aldosterone-regulated sodium reabsorption, bioplanet_458, FoxO family signaling bioplanet_616, regular glucocorticoid receptor pathway bioplanet_1321, mTOR signaling pathway bioplanet_117, interleukin-5 regulation of apoptosis bioplanet_1426, FSH regulation of apoptosis bioplanet_1435, insulin signaling pathway bioplanet_452, interleukin-2 signaling pathway, bioplanet_1421. (**C**) Most studied types of malignant diseases with a SGK1 playing certain role. Analyzed by biostatistics/bioinformatics at MAP Kinase Resource using data curated at Swiss Institute of Bioinformatics (Geneva, Switzeland) (Jonas Cicenas).

There is a lot of evidence that SGK1 is involved in quite a few diseases. Misregulation of SGK1 functions can lead to diseases like hypertension [3,23], inflammation [24,25], fibrosis [26], stroke [27], thrombosis [28], diabetes [3,29] Liddle syndrome [8], autoimmune disorders, pre-cancerous chronic diseases [22], and of course, cancer [30,31] (Figure 3C).

High levels of SGK1 expression have been observed in several tumors including colon cancer [32], prostate cancer [33], ovarian cancer [34], non-small cell lung cancer [35], hepatocellular carcinoma [36], glioblastoma multiforme [5], myeloma [37], and medulloblastoma [38]. On the other hand, SGK1 quantity is known to be down-regulated in several tumors, such as prostate cancer [39], ovarian cancer [40], and adenomatous polyposis coli [41].

Currently used biomarkers could be grouped into risk assessment markers (also known as diagnostic), prognostic markers, and predictive markers. By testing for mutations or other abnormalities in known genes, it is possible to identify individuals who are at increased risk of developing cancer. Identification of these patients has the potential to result in early diagnosis and possibly prevention. Prognostic markers can be characterized as factors which correlate with patient outcome. They are most useful for identification of either patients with favorable outcome which do not require adjuvant systemic therapy or patients with prognosis too poor for conventional approaches [42]. Predictive markers can be defined as factors which predict response or resistance to the specific therapy.

Because of quite an impressive involvement of SGK1 in the development and growth of various tumors, it would be logical to predict, that this kinase is a potential diagnostic, prognostic, or predictive biomarker in cancers. The potency of SGK1 as a biomarker was already shown in several studies.

Fifteen cortisol-secreting adrenocortical adenomas were analyzed together with matched blood samples using high-resolution single nucleotide polymorphism microarrays in order to detect copy number alterations and copy-neutral losses. Forty six recurrent copy number alterations were identified that each affected a single gene (31 gains and 15 losses), including SGK1. CN losses in *SGK1* gene were also confirmed by FISH and DNA qRT-PCR analysis and corresponded to a low mRNA expression level [43]. Another study was performed on 227 adrenocortical tumors (40 adenomas and 187 carcinomas) and 25 normal adrenal tissues using immunohistochemistry and/or tissue microarrays. In addition, 62 frozen tumor samples were used for mRNA analysis. It was found that SGK1 mRNA levels were lower in cortisol-secreting than in nonsecreting tumors (*p* < 0.005). Nonsecreting cancers displayed a significant correlation between SGK1 and CTNNB1 mRNA levels (*p* < 0.001; r = 0.57). Overall survival was shorter in patients with low SGK1 protein expression (HR = 2; 95% CI = 1.24–3.24; *p* = 0.0048). However, disease-free survival was not significant, although with promising tendency (HR = 1.98; 95% CI = 0.9–4.3; *p* = 0.08). Subgroup of patients with a low SGK1 combined with high nuclear β-catenin protein expression showed poor prognosis (HR = 3.3; 95% CI = 0.5–7.3; *p* = 0.03) [44]. The cell culture experiments, using 21 breast cancer cell lines had shown that sensitivity of breast cancer cell lines to AKT inhibitors AZD5363 and MK-2206 correlates with SGK1 mRNA levels. Moreover, knockdown of SGK1 impairs proliferation of AKT-inhibitor-resistant but not -sensitive cells. That effect can be rescued by ectopic SGK1 overexpression [45]. In 224 patient samples and 103 matched adjacent of non-small cell lung cancer samples, it was first established that expression of SGK1 mRNA in cancerous tissues was way higher compared to the adjacent non-cancerous tissues (*p* < 0.001). Interestingly, high SGK1 protein expression was significantly associated with differentiation (χ^2^ = 5.279, *p* = 0.022) and histological type (χ^2^ = 4.127, *p* = 0.042). High expression of SGK1 (HR = 1.926; 95% CI = 1.452–2.903; *p* < 0.001) was a significant negative prognostic factor for five-year survival. In addition, multivariate Cox regression analysis demonstrated that high expression of SGK1 (HR = 1.726; 95% CI = 1.396–2.865; *p* < 0.001) is an independent prognostic factor for the five-year survival [46]. (Table 1)

## 3. SGK1 as Drug Target

**Figure 4:**
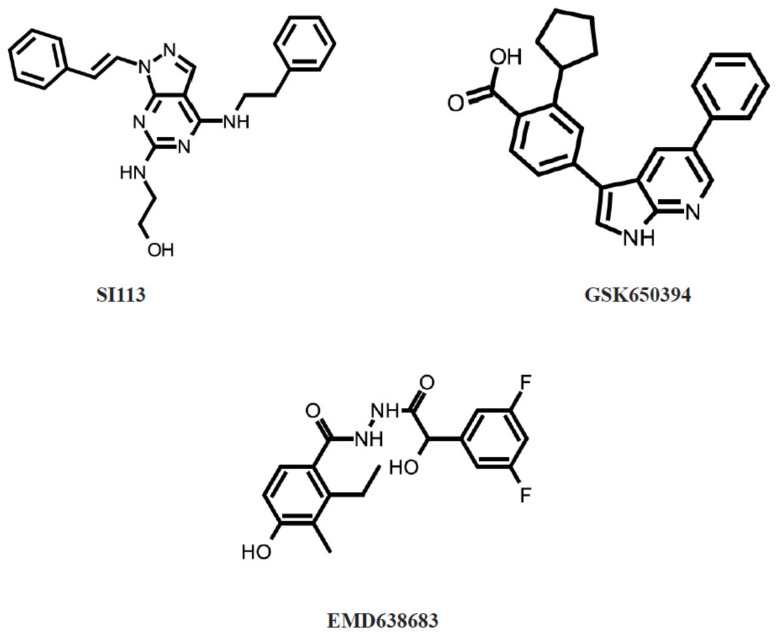
SGK1 inhibitors.

Since abnormalities in SGK1 expression, activity, and regulation have also been found in pathological conditions, search for small-molecule SGK1 inhibitors (Figure 4) for the therapeutic purposes was rigorously initiated and is still ongoing.

*SI113* is a selective SGK1 inhibitor, which has an IC50 of 600 nM (Figure 3). Treatment of RKO human colon carcinoma cell line with 12.5 µM SI113 for 24 h showed an impressive delay in cell cycle progression and accumulation in G0-G1, when compared with untreated cells (*p* = 0.0024). Moreover, combination of SI113 (12.5 µM) with paclitaxel (50 nM) significantly increased apoptosis and necrosis in RKO cells (*p* = 0.00037) [47]. SI113 was also capable to inhibit cell cycle progression and induce apoptosis in HuH-7 and HepG2 hepatocellular carcinoma cell lines. In addition, NOD/SCID female mice were implanted with HuH-7 cells for in vivo treatment with SI113 and showed a significantly smaller tumor volume than control mice (*p* = 0.0009). SI113 also potentiated and synergized with radiotherapy in tumor killing by reducing MDM2 phosphorylation on serine 166 by SGK1 [48]. In glioblastoma multiforme cell lines, significant increase in caspase-mediated apoptosis was detected (*p* < 0.05). It also found that SI113 was co-inducing proliferation inhibition as well as cell cycle block together with radiotherapy, oxidative-stress-mediated cell viability, and autophagy [5]. In endometrial cancer cells, SI113 induced apoptosis, as proven by the cleavage of the apoptotic markers PARP and Caspase-9. It also induced autophagy, which was shown by the increase of the autophagy markers LC3B-II and beclin I. The effects were associated with the induction of endoplasmic reticulum stress markers GRP78 and CHOP [49]. SI113 restores sensitivity to taxanes in tumors resistant or made-resistant to taxanes [59] This inhibitor, in synergy with radionuclides, induces theranostic effects in glioblastoma [60].

*GSK650394* is a selective SGK1 inhibitor, which has an IC50 of 62 nM (Figure 3). Since SGK1 expression was increased under hypoxia and regulated unsaturated fatty acid uptake in NCI-H460 lung adenocarcinoma cells, GSK650394 was applied in order to investigate this effect. GSK650394 caused reduction in fatty acid uptake, decreased long-term survival, and sensitized to the cytotoxic effects of ionizing radiation (*p* ≤ 0.05) [50]. The analog of GSK650394, QGY-5-114-A, inhibited HT29 cell proliferation (*p* < 0.001) and HCT116 cell migration in vitro (*p* < 0.001). This inhibitor also obstructed colonic tumor growth and HCT116 cell proliferation in vivo (*p* < 0.05) [51]. In prostate cancer PC3 and LNCaP cells, GSK650394 impaired migration and invasion in vitro (*p* < 0.05). In addition, GSK650394 treatment also induced autophagy, which led to the inhibition of cell metastasis (*p* < 0.05) [52]. Stimulation of non-small cell lung cancer cell with γ-radiation and GSK650394 induced apoptosis and p53 pathway [46]. Using the subcutaneous xenotransplant mouse model of colorectal cancer (HCT116 and HT29 cells), it was shown that GSK650394 repressed tumor cell proliferation (*p* < 0.05) and tumor growth (*p* < 0.001). In addition, GSK650394 reduced SGK1 expression and increased p27 expression levels in xenograft tumors (*p* < 0.05) [53]. In a study, mice injected with cervical cancer xenograft (ME180 cells) GSK650394 in combination with melatonin, showed a significant tumor size decrease in all cases and even complete tumor remission in 33% of mice (*p* ≤ 0.001) [54]. Inhibition of SGK1 with GSK650394 reduced the radioresistance of colorectal cancer in xenotransplant mouse of HT29 cells. The combination of inhibitor with radiotherapy resulted in minimal tumor size compared to radiotherapy or inhibitor alone (*p* < 0.05) or control groups (*p* < 0.01) [55]. Combined PDGFR inhibitor (CP-673451) and GSK-650394 treatment of xenotransplantation breast cancer models (MDA-MB-231 and BT-549) showed significantly decreased tumor formation compared to control or single agent treatment [61].

*EMD638683* is a highly selective inhibitor of SGK1 kinase, which has an IC50 of 3 μM (Figure 3). In vitro, EMD638683 treatment of colon cancer CaCo-2 cells significantly increased the radiation-induced decrease of forward scatter, increase of phosphatidylserine exposure, decrease of mitochondrial potential, increase of caspase 3 activity, increase of DNA fragmentation, and increase of late apoptosis (*p* < 0.01). In vivo, the number of developing tumors following chemical carcinogenic treatment was significantly blunted by EMD638683 treatment [56]. In breast cancer MCF-7 cells, EMD638683, GSK650394, and testosterone albumin conjugate induced strong apoptotic response and caspase 3 activation (*p* < 0.01), enhanced radiation-induced cell growth control (*p* < 0.001) and induced late FAK and AKT dephosphorylation (*p* < 0.01) [57]. EMD638683 treatment resulted in a statistically significant decline of cell viability in both RD and RH30 rhabdomyosarcoma cells (*p* < 0.05). In addition, doxorubicin treatment decreased the viability of RD and RH30 cells, which was significantly enhanced by the synchronous administration of EMD638683 (*p* < 0.05). The migration and clonogenic potential of RD and RH30 cells were significantly decreased in the presence of EMD638683 (*p* < 0.05) [58].

## 4. Conclusions and Future Perspectives

Cancer biomarker research has developed enormously over the last 15 years, promising new possibilities for cancer diagnostics. Nonetheless, most of the discovered biomarkers have some limitations such as deficiency of methodological standardization, quality control, or significant comparison between healthy individuals and cancer patients. At the moment, all of experimentally discovered biomarkers still need careful validation before they could be applied in everyday diagnostics. Same rules also apply for SGK1; therefore, a lot more pre-clinical and clinical research are needed in order to figure out whether SGK1 is suitable for diagnostics. On the other hand, the rise of “omics” techniques such as genomics, transcriptomics, proteomics, phophoproteomics, kinomics, and metabolomics provide more sensitive detection of biomarkers [42] and allows the use of entire sets of genes instead of just several or sometimes even one biomarker. The combination of different “omics” techniques allows further combination of different types of biomarkers (for example proteins + metabolites + mutated DNA) [62].

In the past decade, developments in the small-molecule SGK1 kinase inhibitor field have led to several products and many more are still in development. Pre-clinical studies in cell lines and animal models provide basal information for the arrangement of clinical studies evaluating the efficiency and side effects of these agents. Clinical studies with SGK1 kinase inhibitors will help to determining which of these inhibitors are most effective for anticancer therapy. Many types of cancers can be targeted by SGK1 inhibitors; thus, many more clinical trials are needed based on pre-clinical findings. Recent increase in anti-kinase small-molecule inhibitors or monoclonal antibodies is already approved by the U.S. Food and Drug Administration, suggesting some perspectives and hopes for SGK1 [63].

In conclusion, the future in SGK1 research both as biomarker and drug target is in our hands and should definitely not be disregarded.

## Figures and Tables

**Table 1:** Most significant studies on SGK1 biomarker and drug target potential.

Cancer Type	Experiment Performed	Significance	Reference
Adrenocortical adenoma	single nucleotide polymorphism microarrays, FISH, DNA qRT-PCR	31 gains and 15 losses of gene detected	[43]
Adrenocortical tumor	immunohistochemistry, tissue microarrays.	Survival was shorter in patients with low SGK1 protein expression (HR = 2; 95% CI = 1.24–3.24; *p* = 0.0048).	[44]
Breast cancer	qRT-PCR	SGK1 mRNA is higher in AKT-inhibitor-resistant cells	[45]
Non-small cell lung cancer	tissue microarray immunohistochemistry, GSK650394 treatment	high expression of SGK1 (HR = 1.726; 95% CI = 1.396–2.865; *p* < 0.001) is an independent prognostic factor for the five year survival. GSK650394 induced apoptosis and p53 pathway.	[46]
Colon carcinoma	SI113 treatment	Cell cycle progression delay compared with untreated cells (*p* = 0.0024).	[47]
Hepatocellular carcinoma	SI113 treatment	Smaller tumor volume than control mice (*p* = 0.0009)	[48]
Glioblastoma multiforme	SI113 treatment	in cell lines, significant increase in caspase-mediated apoptosis were detected (*p* < 0.05).	[5]
Endometrial cancer	SI113 treatment	Apoptosis and autophagy were induced by inhibitor.	[49]
Lung adenocarcinoma	GSK650394 treatment	Decreased long-term survival and sensitized to the cytotoxic effects of ionizing radiation (*p* ≤ 0.05)	[50]
Colon cancer	GSK650394 treatment	inhibitor also obstructed colonic tumor growth and HCT116 cell proliferation in vivo (*p* < 0.05).	[51]
Prostate cancer	GSK650394 treatment	Induced autophagy, which led to the inhibition of cell metastasis (*p* < 0.05).	[52]
Colorectal cancer	GSK650394 treatment	Repressed tumor cell proliferation (*p* < 0.05) and tumor growth (*p* < 0.001).	[53]
Cervical cancer	GSK650394 treatment	GSK650394 in combination with melatonin, caused significant tumor size decrease in all cases and even complete tumor remission in 33% of mice (*p* ≤ 0.001).	[54]
Colorectal cancer	GSK650394 treatment	Combination of inhibitor with radiotherapy resulted in minimal tumor size compared with radiotherapy or inhibitor alone (*p* < 0.05) or control groups (*p* < 0.01).	[55]
Colon cancer	EMD638683 treatment	Number of developing tumors was significantly blunted by EMD638683 treatment.	[56]
Breast cancer	EMD638683 treatment	GSK650394 and testosterone albumin conjugate induced strong apoptotic response and caspase 3 activation (*p* < 0.01), enhanced radiation-induced cell growth control (*p* < 0.001).	[57]
Rhabdomyosarcoma	EMD638683 treatment	Statistically significant decline of cell viability and migration (*p* < 0.05).	[58]

## References

[1] Firestone G.L., Giampaolo J.R., O’Keeffe B.A. (2003). Stimulus-dependent regulation of serum and glucocorticoid inducible protein kinase (SGK) transcription, subcellular localization and enzymatic activity. Cell. Physiol. Biochem. Int. J. Exp. Cell. Physiol. Biochem. Pharmacol..

[2] Waldegger S., Barth P., Raber G., Lang F. (1997). Cloning and characterization of a putative human serine/threonine protein kinase transcriptionally modified during anisotonic and isotonic alterations of cell volume. Proc. Natl. Acad. Sci. USA.

[3] Lang F., Gorlach A., Vallon V. (2009). Targeting SGK1 in diabetes. Expert Opin. Ther. Targets.

[4] Notch E.G., Chapline C., Flynn E., Lameyer T., Lowell A., Sato D., Shaw J.R., Stanton B.A. (2012). Mitogen activated protein kinase 14-1 regulates serum glucocorticoid kinase 1 during seawater acclimation in Atlantic killifish, Fundulus heteroclitus. Comparative biochemistry and physiology. Part A Mol. Integr. Physiol..

[5] Talarico C., Dattilo V., D’Antona L., Barone A., Amodio N., Belviso S., Musumeci F., Abbruzzese C., Bianco C., Trapasso F. (2016). SI113, a SGK1 inhibitor, potentiates the effects of radiotherapy, modulates the response to oxidative stress and induces cytotoxic autophagy in human glioblastoma multiforme cells. Oncotarget.

[6] Hornbeck P.V., Zhang B., Murray B., Kornhauser J.M., Latham V., Skrzypek E. (2015). PhosphoSitePlus, 2014: Mutations, PTMs and recalibrations. Nucleic Acids Res..

[7] Lang F., Shumilina E. (2013). Regulation of ion channels by the serum- and glucocorticoid-inducible kinase SGK1. FASEB J. Off. Publ. Fed. Am. Soc. Exp. Biol..

[8] Faletti C.J., Perrotti N., Taylor S.I., Blazer-Yost B.L. (2002). *sgk*: An essential convergence point for peptide and steroid hormone regulation of ENaC-mediated Na^+^ transport. Am. J. Physiol.-Cell Physiol..

[9] Amato R., D’Antona L., Porciatti G., Agosti V., Menniti M., Rinaldo C., Costa N., Bellacchio E., Mattarocci S., Fuiano G. (2009). Sgk1 activates MDM2-dependent p53 degradation and affects cell proliferation, survival, and differentiation. J. Mol. Med..

[10] Lyo D., Xu L., Foster D.A. (2010). Phospholipase D stabilizes HDM2 through an mTORC2/SGK1 pathway. Biochem. Biophys. Res. Commun..

[11] Amato R., Scumaci D., D’Antona L., Iuliano R., Menniti M., Di Sanzo M., Faniello M.C., Colao E., Malatesta P., Zingone A. (2013). Sgk1 enhances RANBP1 transcript levels and decreases taxol sensitivity in RKO colon carcinoma cells. Oncogene.

[12] Dattilo V., D’Antona L., Talarico C., Capula M., Catalogna G., Iuliano R., Schenone S., Roperto S., Bianco C., Perrotti N. (2017). SGK1 affects RAN/RANBP1/RANGAP1 via SP1 to play a critical role in pre-miRNA nuclear export: A new route of epigenomic regulation. Sci. Rep..

[13] Schmidt E.M., Gu S., Anagnostopoulou V., Alevizopoulos K., Föller M., Lang F., Stournaras C. (2012). Serum- and glucocorticoid-dependent kinase-1-induced cell migration is dependent on vinculin and regulated by the membrane androgen receptor. FEBS J..

[14] Chen L., Wei T.Q., Wang Y., Zhang J., Li H., Wang K.J. (2012). Simulated bladder pressure stimulates human bladder smooth muscle cell proliferation via the PI3K/SGK1 signaling pathway. J. Urol..

[15] Schmid E., Gu S., Yang W., Münzer P., Schaller M., Lang F., Stournaras C., Shumilina E. (2012). Serum- and glucocorticoid-inducible kinase SGK1 regulates reorganization of actin cytoskeleton in mast cells upon degranulation. Am. J. Physiol.-Cell Physiol..

[16] Rotte A., Mack A.F., Bhandaru M., Kempe D.S., Beier N., Scholz W., Dicks E., Potzsch S., Kuhl D., Lang F. (2009). Pioglitazone induced gastric acid secretion. Cell. Physiol. Biochem. Int. J. Exp. Cell. Physiol. Biochem. Pharmacol..

[17] Lang F., Bohmer C., Palmada M., Seebohm G., Strutz-Seebohm N., Vallon V. (2006). (Patho) physiological significance of the serum- and glucocorticoid-inducible kinase isoforms. Physiol. Rev..

[18] Andres-Mateos E., Brinkmeier H., Burks T.N., Mejias R., Files D.C., Steinberger M., Soleimani A., Marx R., Simmers J.L., Lin B. (2013). Activation of serum/glucocorticoid-induced kinase 1 (SGK1) is important to maintain skeletal muscle homeostasis and prevent atrophy. EMBO Mol. Med..

[19] Lang F., Vallon V. (2012). Serum- and glucocorticoid-inducible kinase 1 in the regulation of renal and extrarenal potassium transport. Clin. Exp. Nephrol..

[20] Faresse N., Lagnaz D., Debonneville A., Ismailji A., Maillard M., Fejes-Toth G., Náray-Fejes-Tóth A., Staub O. (2012). Inducible kidney-specific Sgk1 knockout mice show a salt-losing phenotype. Am. J. Physiol.-Ren. Physiol..

[21] Ghani M.J. (2022). SGK1, autophagy and cancer: An overview. Mol. Biol. Rep..

[22] Wu C., Chen Z., Xiao S., Thalhamer T., Madi A., Han T., Kuchroo V. (2018). SGK1 Governs the Reciprocal Development of Th17 and Regulatory T Cells. Cell Rep..

[23] Norlander A.E., Saleh M.A., Pandey A.K., Itani H.A., Wu J., Xiao L., Kang J., Dale B.L., Goleva S.B., Laroumanie F. (2017). A salt-sensing kinase in T lymphocytes, SGK1, drives hypertension and hypertensive end-organ damage. JCI Insight.

[24] Kleinewietfeld M., Manzel A., Titze J., Kvakan H., Yosef N., Linker R.A., Muller D.N., Hafler D.A. (2013). Sodium chloride drives autoimmune disease by the induction of pathogenic TH17 cells. Nature.

[25] Spagnuolo R., Dattilo V., D’Antona L., Cosco C., Tallerico R., Ventura V., Conforti F., Camastra C., Mancina R.M., Catalogna G. (2018). Deregulation of SGK1 in Ulcerative Colitis: A Paradoxical Relationship Between Immune Cells and Colonic Epithelial Cells. Inflamm. Bowel Dis..

[26] Wang H.R., Chen D.L., Zhao M., Shu S.W., Xiong S.X., Gan X.D., Chao S.P., Cao J.L. (2012). C-reactive protein induces interleukin-6 and thrombospondin-1 protein and mRNA expression through activation of nuclear factor-ĸB in HK-2 cells. Kidney Blood Press. Res..

[27] Dahlberg J., Smith G., Norrving B., Nilsson P., Hedblad B., Engström G., Lövkvist H., Carlson J., Lindgren A., Melander O. (2011). Genetic variants in serum and glucocortocoid regulated kinase 1, a regulator of the epithelial sodium channel, are associated with ischaemic stroke. J. Hypertens..

[28] Borst O., Schmidt E.M., Münzer P., Schönberger T., Towhid S.T., Elvers M., Leibrock C., Schmid E., Eylenstein A., Kuhl D. (2012). The serum- and glucocorticoid-inducible kinase 1 (SGK1) influences platelet calcium signaling and function by regulation of Orai1 expression in megakaryocytes. Blood.

[29] Schwab M., Lupescu A., Mota M., Mota E., Frey A., Simon P., Mertens P.R., Floege J., Luft F., Asante-Poku S. (2008). Association of SGK1 gene polymorphisms with type 2 diabetes. Cell. Physiol. Biochem. Int. J. Exp. Cell. Physiol. Biochem. Pharmacol..

[30] Cicenas J., Zalyte E., Rimkus A., Dapkus D., Noreika R., Urbonavicius S. (2017). JNK, p38, ERK, and SGK1 Inhibitors in Cancer. Cancers.

[31] Sang Y., Kong P., Zhang S., Zhang L., Cao Y., Duan X., Sun T., Tao Z., Liu W. (2021). SGK1 in Human Cancer: Emerging Roles and Mechanisms. Front. Oncol..

[32] Lang F., Perrotti N., Stournaras C. (2010). Colorectal carcinoma cells—Regulation of survival and growth by SGK1. Int. J. Biochem. Cell Biol..

[33] Szmulewitz R.Z., Chung E., Al-Ahmadie H., Daniel S., Kocherginsky M., Razmaria A., Zagaja G.P., Brendler C.B., Stadler W.M., Conzen S.D. (2012). Serum/glucocorticoid-regulated kinase 1 expression in primary human prostate cancers. Prostate.

[34] Melhem A., Yamada S.D., Fleming G.F., Delgado B., Brickley D.R., Wu W., Kocherginsky M., Conzen S.D. (2009). Administration of glucocorticoids to ovarian cancer patients is associated with expression of the anti-apoptotic genes SGK1 and MKP1/DUSP1 in ovarian tissues. Clin. Cancer Res. Off. J. Am. Assoc. Cancer Res..

[35] Abbruzzese C., Mattarocci S., Pizzuti L., Mileo A.M., Visca P., Antoniani B., Alessandrini G., Facciolo F., Amato R., D’Antona L. (2012). Determination of SGK1 mRNA in non-small cell lung cancer samples underlines high expression in squamous cell carcinomas. J. Exp. Clin. Cancer Res. CR.

[36] Chung E.J., Sung Y.K., Farooq M., Kim Y., Im S., Tak W.Y., Hwang Y.J., Kim Y.I., Han H.S., Kim J.C. (2002). Gene expression profile analysis in human hepatocellular carcinoma by cDNA microarray. Mol. Cells.

[37] Fagerli U.M., Ullrich K., Stühmer T., Holien T., Köchert K., Holt R.U., Bruland O., Chatterjee M., Nogai H., Lenz G. (2011). Serum/glucocorticoid-regulated kinase 1 (SGK1) is a prominent target gene of the transcriptional response to cytokines in multiple myeloma and supports the growth of myeloma cells. Oncogene.

[38] Yoon J.W., Gilbertson R., Iannaccone S., Iannaccone P., Walterhouse D. (2009). Defining a role for Sonic hedgehog pathway activation in desmoplastic medulloblastoma by identifying GLI1 target genes. Int. J. Cancer.

[39] Rauhala H.E., Porkka K.P., Tolonen T.T., Martikainen P.M., Tammela T.L., Visakorpi T. (2005). Dual-specificity phosphatase 1 and serum/glucocorticoid-regulated kinase are downregulated in prostate cancer. Int. J. Cancer.

[40] Chu S., Rushdi S., Zumpe E.T., Mamers P., Healy D.L., Jobling T., Burger H.G., Fuller P.J. (2002). FSH-regulated gene expression profiles in ovarian tumours and normal ovaries. Mol. Hum. Reprod..

[41] Segditsas S., Sieber O., Deheragoda M., East P., Rowan A., Jeffery R., Nye E., Clark S., Spencer-Dene B., Stamp G. (2008). Putative direct and indirect Wnt targets identified through consistent gene expression changes in APC-mutant intestinal adenomas from humans and mice. Hum. Mol. Genet..

[42] Ger M., Kaupinis A., Petrulionis M., Kurlinkus B., Cicenas J., Sileikis A., Valius M., Strupas K. (2018). Proteomic Identification of FLT3 and PCBP3 as Potential Prognostic Biomarkers for Pancreatic Cancer. Anticancer Res..

[43] Ronchi C.L., Leich E., Sbiera S., Weismann D., Rosenwald A., Allolio B., Fassnacht M. (2012). Single nucleotide polymorphism microarray analysis in cortisol-secreting adrenocortical adenomas identifies new candidate genes and pathways. Neoplasia.

[44] Ronchi C.L., Sbiera S., Leich E., Tissier F., Steinhauer S., Deutschbein T., Fassnacht M., Allolio B. (2012). Low SGK1 expression in human adrenocortical tumors is associated with ACTH-independent glucocorticoid secretion and poor prognosis. J. Clin. Endocrinol. Metab..

[45] Sommer E.M., Dry H., Cross D., Guichard S., Davies B.R., Alessi D.R. (2013). Elevated SGK1 predicts resistance of breast cancer cells to Akt inhibitors. Biochem. J..

[46] Tang Z., Shen Q., Xie H., Zhou Z., Shi G., Zhang C., Mohammed A., Wu Y., Ni S., Zhou X. (2018). Serum and glucocorticoid-regulated kinase 1 (SGK1) is a predictor of poor prognosis in non-small cell lung cancer, and its dynamic pattern following treatment with SGK1 inhibitor and γ-ray irradiation was elucidated. Oncol. Rep..

[47] D’Antona L., Amato R., Talarico C., Ortuso F., Menniti M., Dattilo V., Iuliano R., Gigliotti F., Artese A., Costa G. (2015). SI113, a specific inhibitor of the Sgk1 kinase activity that counteracts cancer cell proliferation. Cell. Physiol. Biochem. Int. J. Exp. Cell. Physiol. Biochem. Pharmacol..

[48] Talarico C., D’Antona L., Scumaci D., Barone A., Gigliotti F., Fiumara C.V., Dattilo V., Gallo E., Visca P., Ortuso F. (2015). Preclinical model in HCC: The SGK1 kinase inhibitor SI113 blocks tumor progression in vitro and in vivo and synergizes with radiotherapy. Oncotarget.

[49] Conza D., Mirra P., Calì G., Tortora T., Insabato L., Fiory F., Schenone S., Amato R., Beguinot F., Perrotti N. (2017). The SGK1 inhibitor SI113 induces autophagy, apoptosis, and endoplasmic reticulum stress in endometrial cancer cells. J. Cell. Physiol..

[50] Matschke J., Wiebeck E., Hurst S., Rudner J., Jendrossek V. (2016). Role of SGK1 for fatty acid uptake, cell survival and radioresistance of NCI-H460 lung cancer cells exposed to acute or chronic cycling severe hypoxia. Radiat. Oncol..

[51] Liang X., Lan C., Zhou J., Fu W., Long X., An Y., Jiao G., Wang K., Li Y., Xu J. (2017). Development of a new analog of SGK1 inhibitor and its evaluation as a therapeutic molecule of colorectal cancer. J. Cancer.

[52] Liu W., Wang X., Wang Y., Dai Y., Xie Y., Ping Y., Yin B., Yu P., Liu Z., Duan X. (2018). SGK1 inhibition-induced autophagy impairs prostate cancer metastasis by reversing EMT. J. Exp. Clin. Cancer Res. CR.

[53] Liang X., Lan C., Jiao G., Fu W., Long X., An Y., Wang K., Zhou J., Chen T., Li Y. (2017). Therapeutic inhibition of SGK1 suppresses colorectal cancer. Exp. Mol. Med..

[54] Wang M., Xue Y., Shen L., Qin P., Sang X., Tao Z., Yi J., Wang J., Liu P., Cheng H. (2019). Inhibition of SGK1 confers vulnerability to redox dysregulation in cervical cancer. Redox Biol..

[55] Zhou C., Xiao W., Jiang T., Guo Z., Li M., Chang H., Wu Y., Chen M., Shi M., Xu W. (2020). Targeting SGK1 enhances the efficacy of radiotherapy in locally advanced rectal cancer. Biomed. Pharmacother..

[56] Towhid S.T., Liu G.L., Ackermann T.F., Beier N., Scholz W., Fuchß T., Toulany M., Rodemann H.P., Lang F. (2013). Inhibition of Colonic Tumor Growth by the Selective SGK Inhibitor EMD638683. Cell. Physiol. Biochem..

[57] Liu G., Honisch S., Liu G., Schmidt S., Pantelakos S., Alkahtani S., Toulany M., Lang F., Stournaras C. (2015). Inhibition of SGK1 enhances mAR-induced apoptosis in MCF-7 breast cancer cells. Cancer Biol. Ther..

[58] Schmid E., Stagno M.J., Yan J., Schleicher S., Yu W., Honisch S., Lang F., Fuchs J., Seitz G. (2017). Serum and Glucocorticoid Inducible Kinase 1-Sensitive Survival, Proliferation and Migration of Rhabdomyosarcoma Cells. Cell. Physiol. Biochem. Int. J. Exp. Cell. Physiol. Biochem. Pharmacol..

[59] D’Antona L., Dattilo V., Catalogna G., Scumaci D., Fiumara C.V., Musumeci F., Perrotti G., Schenone S., Tallerico R., Spoleti C.B. (2019). In Preclinical Model of Ovarian Cancer, the SGK1 Inhibitor SI113 Counteracts the Development of Paclitaxel Resistance and Restores Drug Sensitivity. Transl. Oncol..

[60] Catalogna G., Talarico C., Dattilo V., Gangemi V., Calabria F., D’Antona L., Schenone S., Musumeci F., Bianco C., Perrotti N. (2017). The SGK1 Kinase Inhibitor SI113 Sensitizes Theranostic Effects of the 64CuCl2 in Human Glioblastoma Multiforme Cells. Cell. Physiol. Biochem. Int. J. Exp. Cell. Physiol. Biochem. Pharmacol..

[61] Yang L., Li N., Xue Z., Liu L.R., Li J., Huang X., Xie X., Zou Y., Tang H., Xie X. (2020). Synergistic therapeutic effect of combined PDGFR and SGK1 inhibition in metastasis-initiating cells of breast cancer. Cell Death Differ..

[62] Chen F., Wang J., Wu Y., Gao Q., Zhang S. (2022). Potential Biomarkers for Liver Cancer Diagnosis Based on Multi-Omics Strategy. Front. Oncol..

[63] Cicenas J., Račienė A. (2021). Anti-Cancer Drugs Targeting Protein Kinases Approved by FDA in 2020. Cancers.

